# Association of parental prepregnancy BMI with neonatal outcomes and birth defect in fresh embryo transfer cycles: a retrospective cohort study

**DOI:** 10.1186/s12884-021-04261-y

**Published:** 2021-11-27

**Authors:** Ruixue Chen, Lifen Chen, Yifeng Liu, Feixia Wang, Siwen Wang, Yun Huang, Kai-Lun Hu, Yuzhi Fan, Ruoyan Liu, Runjv Zhang, Dan Zhang

**Affiliations:** 1 Key Laboratory of Reproductive Genetics (Ministry of Education), Department of Reproductive Endocrinology, Women’s Hospital, Zhejiang University School of Medicine, Hangzhou, 310006 Zhejiang China; 2 School of Medicine, Zhejiang University, Hangzhou, 310058 Zhejiang China; 3 Women’s Reproductive Health Research Key Laboratory of Zhejiang Province, Women’s Hospital, Zhejiang University School of Medicine, Hangzhou, 310006 Zhejiang China

**Keywords:** Body mass index, Obesity, Assisted reproductive technology, Neonatal outcome, Birth defect

## Abstract

**Background:**

Parental body mass index (BMI) is associated with pregnancy outcomes. But the effect of parental prepregnancy BMI on offspring conceived via in vitro fertilization (IVF) or intracytoplasmic sperm injection (ICSI), especially the birth defect, remains to be determined. This study aimed to investigate the associations of parental prepregnancy BMI with neonatal outcomes and birth defect in fresh embryo transfer cycles.

**Methods:**

We conducted a retrospective cohort study including 5741 couples in their first fresh IVF/ICSI cycles admitted to Women’s Hospital, School of Medicine, Zhejiang University from January 2013 to July 2016. The primary outcome was birth defects, which was classified according to the International Classification of Diseases, 10th Revision. Secondary outcomes included preterm delivery rate, infant gender, birth weight, small-for-gestational age (SGA) and large-for-gestational age (LGA). Multilevel regression analyses were used to assess the associations of parental prepregnancy BMI with neonatal outcomes and birth defect.

**Results:**

In singletons, couples with prepregnancy BMI ≥25 kg/m^2^ had higher odds of LGA than those with BMI < 25 kg/m^2^. The birth defect rate was significantly higher when paternal prepregnancy BMI ≥25 kg/m^2^ in IVF cycles (aOR 1.82, 95% CI 1.06–3.10) and maternal BMI ≥25 kg/m^2^ in ICSI cycles (aOR 4.89, 95% CI 1.45–16.53). For subcategories of birth defects, only the odds of congenital malformations of musculoskeletal system was significantly increased in IVF offspring with paternal BMI ≥25 kg/m^2^ (aOR 4.55, 95% CI 1.32–15.71). For twins, there was no significant difference among four groups, except for the lower birth weight of IVF female infants.

**Conclusions:**

Parental prepregnancy BMI ≥25 kg/m^2^ is associated with higher incidence of LGA in IVF/ICSI singletons. Paternal prepregnancy BMI ≥25 kg/m^2^ was likely to have higher risk of birth defect in IVF offspring than those with BMI < 25 kg/m^2^, particularly in the musculoskeletal system. It is essential for overweight or obesity couples to lose weight before IVF/ICSI treatments.

**Supplementary Information:**

The online version contains supplementary material available at 10.1186/s12884-021-04261-y.

## Introduction

Overweight and obesity are becoming one of the most important worldwide health issue. The prevalence of overweight and obesity has increased globally over the past decades [1]. In China, the proportion of adults with overweight has increased to 34.3% and the proportion of obesity has increased to 16.4%.

Overweight and obesity are associated with the risk of many diseases, such as hypertension, cardiovascular diseases, diabetes and cancer [2–4]. In recent decades, more attention has been paid to the effects of overweight and obesity on the human reproductive function. Accumulating evidence suggest that overweight and obesity can contribute to the poor quality of oocyte and sperm, anovulation and impairment of endometrial receptivity [5–8]. Many studies have investigated the impact of maternal and paternal body mass index (BMI) on assisted reproductive technology (ART) treatment outcomes and neonatal outcomes, independently or combined. However, the results in these studies are not consistent. A retrospective cohort study of 12, 061 first fresh in vitro fertilization (IVF) or intracytoplasmic sperm injection (ICSI) cycles in China found that the singletons’ birth weight increased with parental BMI [9]. Another retrospective analysis of 287, 213 pregnancies in London showed that maternal obesity increased the odds of emergency caesarian section and the large-for-gestational age (LGA) [10]. A meta-analysis found that increased paternal BMI is associated with higher risk of small-for-gestational age (SGA) and macrosomia [11]. Nevertheless, a number of studies observed that paternal BMI had no association with birth weight, LGA and SGA [12, 13].

In addition, birth defect is a global health problem which can result in death or disability of offspring. A lot of studies had discussed different maternal or paternal factors, like advanced age, lifestyle, obesity and chronic diseases, which would improve the birth defect rate of natural pregnancy or pregnancy that does not distinguish between natural and assisted reproduction [14–17]. Some research about the birth defect of ART offspring paid more attention on the effect of different types of ART procedures, such as sperm injection and frozen embryo transfer [18, 19]. However, limited researches have examined the impact of parental prepregnancy BMI on birth defect of only ART offspring.

The current study aimed to estimate the effects of parental prepregnancy BMI on the neonatal outcomes and birth defect of offspring via IVF/ICSI. To the author’s knowledge, this analysis is the first to investigate the association of paternal prepregnancy BMI with the birth defect risk of ART offspring. Analyses were also stratified by conventional IVF or ICSI to investigate potential ART procedures differences.

## Methods

### Study design and participants

This study was approved by the ethnic committee in Women’s Hospital, School of Medicine, Zhejiang University (reference: IRB-20200364-R) and all data were collected from the electronic medical record system in the department of Reproductive Medicine Center. Couples who underwent first IVF/ICSI fresh embryo transfer cycles with autologous oocytes from January 2013 to July 2016 were analyzed. The exclusion criteria were as follows:1) maternal age > 45 years; 2) couples with severe complication before pregnancy, such as diabetes, hypertension, heart or liver disease; 3) couples with a history of smoking or drinking; 4) couples underwent preimplantation genetic testing; 5) data were incomplete or incorrect in the database. A history of smoking was defined as a patient who smoked 1 or more cigarettes per day for at least the previous six months [20]. A history of drinking was defined as 60 or more grams of pure alcohol on at least one single occasion in the past seven days [21].

### Parental prepregnancy BMI

Prepregnancy weight and height of all couples were measured by a trained nurse. BMI was calculated as weight divided by squared height. All 5741 couples were divided into four groups based on the parental prepregnancy BMI according to the classification criteria of the World Health Organization: group A (both maternal and paternal BMI < 25 kg/m^2^); group B (maternal BMI < 25 kg/m^2^ and paternal BMI ≥25 kg/m^2^); group C (maternal BMI ≥25 kg/m^2^ and paternal BMI < 25 kg/m^2^); group D (both maternal and paternal BMI ≥25 kg/m^2^).

### Outcomes

The primary outcome was birth defect, which was determined by birth hospital or pediatric care center and was followed by a trained nurse up to the 3 years old of the child. It was classified according to the International Classification of Diseases, 10th Revision (ICD-10) into 9 subcategories. Two additional categories, ‘any birth defect’ and ‘multiple birth defects’, were used in our study. Any birth defect was defined as at least one subcategory of birth defects and multiple birth defects were defined as more than one subcategory of birth defects.

Secondary outcomes included live birth rate, delivery method, preterm delivery rate, infant gender, birth weight, low birth weight (birth weight < 2500 g), macrosomia (birth weight ≥ 4000 g), SGA and LGA. Live birth was defined as at least one live infant born at 28 weeks or more of gestation. Delivery methods include cesarean section and vaginal delivery. Preterm delivery was defined as a delivery occurring before 37 gestational weeks. The definition of SGA was the birth weight less than the 10th percentile for the gestational age and sex. LGA was defined as weighing greater than 90th percentile for the gestational age and sex. The 2014 reference of Chinese infants from 28 to 44 gestation weeks was used as the reference in this study [22].

### Covariates

Covariates included: parental age (in years), reason for ART (ovulatory dysfunction, diminished ovarian reserve, endometriosis/tubal factor, uterine factor, male factor, unexplained), primary infertility or secondary infertility, duration of infertility (in years), number of oocytes retrieved, IVF or ICSI, number of two pronuclear (2PN) zygotes, number of embryos transferred.

### Statistical analyses

SPSS statistics 22.0 (IBM) was used for all statistical data analyses. All continuous variables, such as parental age, BMI and birth weight were presented as the mean ± SD and compared by means of analysis of variance. Categorical variables, such as live birth, LGA, SGA and birth defect, were presented as frequencies and percentages and compared by means of chi-square tests. Fisher’s exact test was used if the number of cycles or participants in one or more categories was less than five.

Subgroup analyses was performed by the conventional IVF or ICSI and the singletons or twin delivery. Multilevel logistic regression was used to explore the effect of parental prepregnancy BMI on live birth, mode of delivery, infant gender, low birth weight, macrosomia, SGA, LGA and any birth defect, which were described as odds ratio (OR) with 95% confidence intervals (CIs). Multilevel linear regression analyses were used to investigate the association between parental prepregnancy BMI and birth weight, which was described as unstandardized regression coefficient (B) with 95% CIs. All analyses were adjusted for parental age, type of infertility, duration of infertility, ovulatory dysfunction and endometriosis, which were described as adjusted odds ratio (aOR) or adjusted regression coefficient (aB) with 95% CIs.

Additionally, all IVF cycles was stratified into two groups (paternal prepregnancy BMI < 25 kg/m^2^ and paternal prepregnancy BMI ≥25 kg/m^2^) according to paternal prepregnancy BMI to dig deeper into the effect of paternal prepregnancy BMI on the subcategories of birth defects for singletons conceived via IVF. Logistic regression was used to analyze IVF cycles separately. Analyses were adjusted for parental age, maternal BMI, type of infertility, duration of infertility, ovulatory dysfunction and endometriosis. *P* values < 0.05 was considered statistically significant and all tests were 2-tailed.

## Results

### Characteristics of study population

**Fig. 1:**
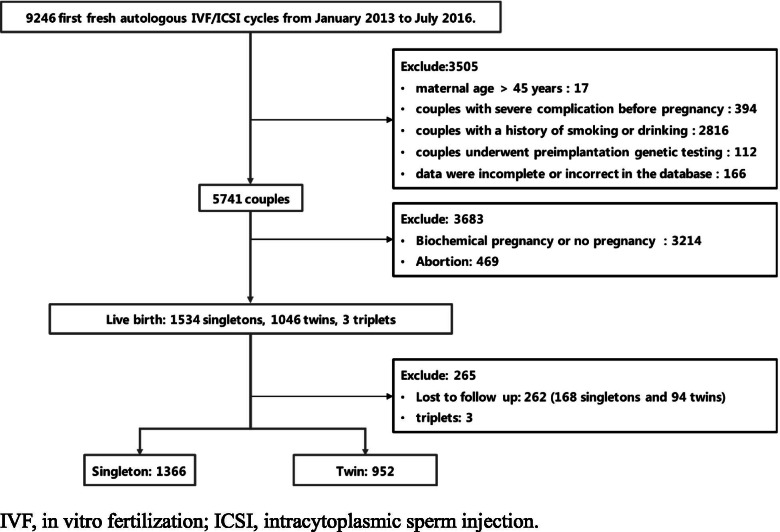
Flowchart of the study population

Among 5741 couples enrolled in this study, 4175 couples (72.7%) underwent IVF treatment and 1566 couples (27.3%) underwent ICSI treatment. The included couples delivered 2583 neonates (1534 singletons, 1046 twins and 3 triplets). 1366 singletons and 952 twins have information about birth defect (Fig. 1).

Table 1 showed baseline characteristics of all included couples compared by parental prepregnancy BMI. The parental age and duration of infertility were significantly higher among group B, C and D compared with group A (all *P* < 0.001). There were significant differences in the proportion of primary infertility, ovulatory dysfunction, endometriosis and ART method across the parental prepregnancy BMI categories (all *P* < 0.05). No difference appeared across the BMI categories when comparing the proportion of diminished ovarian reserve, tubal factor, uterine factor, male factor and unexplained infertility.

**Table 1:** Characteristics of the included couples compared by maternal and paternal prepregnancy BMI

Variable	Prepregnancy BMI category (kg/m^**2**^)				***P*** value ^a^
	A(M&***P*** < 25)	B(M < 25&P ≥ 25)	C(M ≥ 25&P < 25)	D(M&P ≥ 25)	
No. of cycles	3276	1571	533	361	
Female age(y)	30.89 ± 4.50	31.51 ± 4.46	31.39 ± 4.68	32.55 ± 4.74	**<0.001**
Male age(y)	32.83 ± 5.32	33.49 ± 5.41	33.70 ± 5.52	34.96 ± 6.02	**<0.001**
Female BMI(kg/m^2^)	21.23 ± 2.01	21.44 ± 1.99	26.89 ± 1.59	26.99 ± 1.75	**<0.001**
Male BMI(kg/m^2^)	22.09 ± 1.94	27.27 ± 1.96	22.26 ± 1.93	27.42 ± 2.08	**<0.001**
Type of infertility					**0.006**
Primary	1633(49.8)	755(48.1)	228(42.8)	159(44.0)	
Secondary	1643(50.2)	816(51.9)	305(57.2)	202(56.0)	
Duration of infertility(y)	3.72 ± 2.78	4.07 ± 3.13	4.27 ± 3.11	4.34 ± 3.39	**<0.001**
Cause of infertility					
Ovulatory dysfunction	209(6.4)	104(6.6)	84(15.8)	60(16.6)	**<0.001**
Diminished ovarian reserve	248(7.6)	122(7.8)	28(5.3)	27(7.5)	0.258
Endometriosis	453(13.8)	186(11.8)	44(8.3)	27(7.5)	**<0.001**
Tubal factor	2057(62.8)	1015(64.6)	360(67.5)	230(63.7)	0.163
Uterine factor	257(7.8)	113(7.2)	47(8.8)	38(10.5)	0.164
Male factor	1052(32.1)	513(32.7)	154(28.9)	121(33.5)	0.382
Unexplained	230(7.0)	98(6.2)	26(4.9)	23(6.4)	0.275
ART method					**0.045**
IVF	2346(71.6)	1152(73.3)	412(77.3)	265(73.4)	
ICSI	930(28.4)	419(26.7)	121(22.7)	96(26.6)	

Values are presented as mean ± SD or frequency (percentage)

^a^ P value is based on One-Way ANOVA for continuous variables and χ2 test for categorical variables across maternal and paternal prepregnancy BMI. Results in bold indicate statistical significance (*P* < 0.05)

*BMI* body mass index, *M* maternal prepregnancy BMI, *P* paternal prepregnancy BMI, *y* year; *ART* assisted reproductive technology, *IVF* in vitro fertilization, *ICSI* intracytoplasmic sperm injection

### Cycle outcomes

The IVF/ICSI treatment outcomes are summarized in Table S1. No significant difference in the number of oocytes retrieved, 2PN rate, number of embryos transferred and clinical pregnancy rate were observed among four groups. Compared with group A, couples in group B, C and D were more likely to have lower live birth rate (38.1% vs 36.4% vs 31.6% vs 31.7%, *P* = 0.022) when analyzing IVF cycles only. After adjustment for parental age, duration of infertility, type of infertility, women with ovulatory dysfunction and endometriosis, couples with maternal prepregnancy BMI ≥25 kg/m^2^ was associated with lower live birth rate compared with both parental prepregnancy BMI <25 kg/m^2^ (group C vs. group A: aOR 0.79, 95% CI 0.63–0.99).

### Neonatal outcomes

We analyzed the clinical data of 1534 singletons and 1046 twins (Table 2). For singletons, the proportion of female newborns and incidence of cesarean section and fetal macrosomia varied significantly among parental prepregnancy BMI categories in IVF subgroup (*P* < 0.05). No significant difference was observed for birth weight when considering all IVF or ICSI singletons. However, the birth weight of female newborns in IVF subgroup increased significantly with higher BMI categories, ranging from 3.17Kg (SD 0.42) in group A (both parental prepregnancy BMI < 25 kg/m^2^) to 3.35Kg (SD 0.45) in group D (both parental prepregnancy BMI ≥25 kg/m^2^). LGA occurred more frequently in higher parental prepregnancy BMI categories, with the incidence of 16.1% in group B, 21.5% in group C and 20.0% in group D compared to 10.2% in group A for IVF subgroup and the incidence of 26.2% in group C and 31.8% in group D compared to 12.4% in group A for ICSI subgroup. No significant difference in gestational age, premature birth rate, low birth weight infant rate and SGA rate was observed among BMI categories.

**Table 2:** The neonatal outcomes compared by maternal and paternal prepregnancy BMI

Variable	IVF					ICSI				
	A(M&P < 25)	B(M < 25&P ≥ 25)	C(M ≥ 25&P < 25)	D(M&P ≥ 25)	P value ^a^	A(M&P < 25)	B(M < 25&P ≥ 25)	C(M ≥ 25&P < 25)	D(M&P ≥ 25)	***P*** value ^a^
Singletons										
No. of cycles/newborns	668	298	93	60		234	117	42	22	
Cesarean section	401(60.0)	179(60.1)	71(76.3)	40(66.7)	**0.017**	128(54.7)	73(62.4)	31(73.8)	13(59.1)	0.104
Gestational age(wk)	38.61 ± 1.54	38.66 ± 1.58	38.22 ± 2.09	38.68 ± 1.43	0.114	38.62 ± 1.67	38.84 ± 1.38	38.19 ± 2.30	38.50 ± 1.68	0.184
Premature birth	45(6.7)	18(6.0)	10(10 .8)	6(10.0)	0.306	15(6.4)	7(6.0)	5(11.9)	3(13.6)	0.279
Gender					**0.008**					0.268
Boys	375(56.1)	162(54.4)	36(38.7)	27(45.0)		120(51.3)	61(52.1)	24(57.1)	7(31.8)	
Girls	293(43.9)	136(45.6)	57(61.3)	33(55.0)		114(48.7)	56(47.9)	18(42.9)	15(68.2)	
Birth weight(kg)	3.24 ± 0.47	3.31 ± 0.48	3.29 ± 0.60	3.35 ± 0.55	0.098	3.29 ± 0.52	3.34 ± 0.46	3.35 ± 0.70	3.38 ± 0.46	0.699
Birth weight (kg, boys)	3.29 ± 0.49	3.36 ± 0.48	3.27 ± 0.68	3.35 ± 0.66	0.551	3.38 ± 0.46	3.33 ± 0.53	3.36 ± 0.60	3.18 ± 0.30	0.693
Birth weight (kg, girls)	3.17 ± 0.42	3.25 ± 0.48	3.30 ± 0.56	3.35 ± 0.45	**0.031**	3.19 ± 0.56	3.35 ± 0.38	3.34 ± 0.83	3.48 ± 0.49	0.086
Low birth weight infant	35(5.2)	15(5.0)	11(11.8)	2(3.3)	0.086	10(4.3)	5(4.3)	3(7.1)	0	0.713
Fetal macrosomia	34(5.1)	16(5.4)	7(7.5)	9(15.0)	**0.030**	18(7.7)	9(7.7)	8(19.0)	3(13.6)	0.093
SGA	52(7.8)	18(6.0)	6(6.5)	3(5.0)	0.787	16(6.8)	7(6.0)	2(4.8)	2(9.1)	0.909
LGA	68(10.2)	48(16.1)	20(21.5)	12(20.0)	**0.001**	29(12.4)	14(12.0)	11(26.2)	7(31.8)	**0.010**
Twins										
No. of cycles	226	120	37	24		73	34	5	4	
Cesarean section	213(94.2)	116(96.7)	35(94.6)	24(100.0)	0.668	71(97.3)	33(97.1)	5(100.0)	4(100.0)	1.000
Gestational age(wk)	36.11 ± 1.93	35.86 ± 2.10	35.65 ± 2.23	35.58 ± 2.57	0.375	36.37 ± 1.59	36.29 ± 1.77	36.2 ± 1.92	36.50 ± 1.73	0.989
Premature birth	115(50.9)	62(51.7)	21(56.8)	14(58.3)	0.840	34(46.6)	15(44.1)	2(40.0)	1(25.0)	0.906
No. of newborns	452	240	74	48		146	68	10	8	
Gender					0.985					0.829
Boys	230(50.9)	125(52.1)	37(50.0)	25(52.1)		73(50.0)	33(48.5)	4(40.0)	5(62.5)	
Girls	222(49.1)	115(47.9)	37(50.0)	23(47.9)		73(50.0)	35(51.5)	6(60.0)	3(37.5)	
Birth weight(kg)	2.44 ± 0.46	2.38 ± 0.47	2.41 ± 0.59	2.39 ± 0.59	0.366	2.49 ± 0.49	2.46 ± 0.40	2.40 ± 0.42	2.74 ± 0.38	0.382
Birth weight (kg, boys)	2.48 ± 0.49	2.44 ± 0.46	2.63 ± 0.62	2.42 ± 0.58	0.247	2.52 ± 0.48	2.48 ± 0.51	2.69 ± 0.29	2.71 ± 0.48	0.697
Birth weight (kg, girls)	2.41 ± 0.43	2.31 ± 0.48	2.20 ± 0.49	2.36 ± 0.60	**0.031**	2.47 ± 0.50	2.44 ± 0.27	2.20 ± 0.40	2.80 ± 0.22	0.270
Low birth weight infant	218(48.2)	134(55.8)	38(51.4)	17(35.4)	**0.047**	67(45.9)	33(48.5)	6(60.0)	1(12.5)	0.203
Fetal macrosomia	0	0	1(1.4)	0	0.150	1(0.7)	0	0	0	1.000
SGA	153(33.8)	86(35.8)	27(36.5)	10(20.8)	0.233	51(34.9)	28(41.2)	5(50.0)	1(12.5)	0.315
LGA	2(0.4)	0	1(1.4)	0	0.322	1(0.7)	0	1(10.0)	0	0.149

Values are presented as mean ± SD or frequency (percentage)

^a^ *P* value is based on One-Way ANOVA for continuous variables and χ2 test for categorical variables across maternal and paternal prepregnancy BMI. Results in bold indicate statistical significance (*P* < 0.05)

*BMI* body mass index, *M* maternal prepregnancy BMI, *P* paternal prepregnancy BMI, *IVF* in vitro fertilization, *ICSI* intracytoplasmic sperm injection, *wk* week, *SGA* small for gestational age, *LGA* large for gestational age

For twins, except for the birth weight of female newborns and the proportion of low birth weight in IVF subgroup, there was no difference among four BMI categories.

### Association between parental prepregnancy BMI and neonatal outcomes

Table 3 included details on the associations between parental prepregnancy BMI and neonatal outcomes of singletons. For IVF treatments, couples with maternal prepregnancy BMI ≥25 kg/m^2^ had a trend towards an increased risk of cesarean section (aOR 1.98, 95% CI 1.19–3.30) and a two-fold increased risk of low birth weight infant (aOR 2.42, 95% CI 1.16–5.02) compared to couples with both parental prepregnancy BMI < 25 kg/m^2^. Couples with both parental prepregnancy BMI ≥25 kg/m^2^ had a three-fold increased risk of fetal macrosomia (aOR 3.20, 95% CI 1.43–7.18). Higher risk of LGA was observed for couples with maternal and paternal prepregnancy BMI ≥25 kg/m^2^, independent or combined, than both parental BMI < 25 kg/m^2^ (group B: aOR 1.70, 95% CI 1.14–2.54; group C: aOR 2.48, 95% CI 1.41–4.36; group D: aOR 2.27, 95% CI 1.14–4.52). Female singletons from the couples with maternal prepregnancy BMI ≥25 kg/m^2^ had higher birth weight (aB 0.14, 95% CI 0.01–0.27) than both parental BMI < 25 kg/m^2^, and similar trend was observed for couples with both parental prepregnancy BMI ≥25 kg/m^2^ (aB 0.20, 95% CI 0.03–0.36) (Table S2). For ICSI treatments, couples with maternal prepregnancy BMI ≥25 kg/m^2^ had a two-fold increased risk of cesarean section (aOR 2.52, 95% CI 1.18–5.36) and a three-fold higher risk of fetal macrosomia (aOR 3.48, 95% CI 1.32–9.19) compared to couples with both parental prepregnancy BMI < 25 kg/m^2^. Couples with maternal prepregnancy BMI ≥25 kg/m^2^ had a nearly three-fold higher risk of LGA (aOR 2.94, 95% CI 1.27–6.81), for couples with both parental prepregnancy BMI ≥25 kg/m^2^ a similar trend (aOR 3.16, 95% CI 1.48–8.76) was observed.

**Table 3:** Associations between parental prepregnancy BMI and neonatal outcomes of singletons in multilevel logistic regression analyses

Variable	IVF				ICSI			
	A(M&***P*** < 25)	B(M < 25&***P*** ≥ 25)	C(M ≥ 25&***P*** < 25)	D(M&P ≥ 25)	A(M&P < 25)	B(M < 25&P ≥ 25)	C(M ≥ 25&P < 25)	D(M&P ≥ 25)
Cesarean section	401(60.0)	179(60.1)	71(76.3)	40(66.7)	128(54.7)	73(62.4)	31(73.8)	13(59.1)
aOR (95% CI) ^a^	REF	0.96(0.72,1.27)	1.98(1.19,3.30)	1.30(0.73,2.29)	REF	1.37(0.87,2.18)	2.52(1.18,5.36)	1.25(0.51,3.08)
P value ^b^		0.755	**0.008**	0.374		0.177	**0.017**	0.633
Gender(Boys)	375(56.1)	162(54.4)	36(38.7)	27(45.0)	120(51.3)	61(52.1)	24(57.1)	7(31.8)
aOR (95% CI) ^a^	REF	0.93(0.71,1.23)	0.49(0.31,0.77)	0.65(0.38,1.11)	REF	1.02(0.65,1.60)	1.28(0.65,2.51)	0.44(0.17,1.13)
P value ^b^		0.617	**0.002**	0.113		0.923	0.477	0.088
Low birth weight infant	35(5.2)	15(5.0)	11(11.8)	2(3.3)	10(4.3)	5(4.3)	3(7.1)	0
aOR (95% CI) ^a^	REF	0.97(0.52,1.81)	2.42(1.16,5.02)	0.60(0.14,2.61)	REF	1.08(0.35,3.30)	2.10(0.52,8.54)	–
P value ^b^		0.924	**0.018**	0.499		0.897	0.298	–
Fetal macrosomia	34(5.1)	16(5.4)	7(7.5)	9(15.0)	18(7.7)	9(7.7)	8(19.0)	3(13.6)
aOR (95% CI) ^a^	REF	1.05(0.57,1.95)	1.48(0.63,3.48)	3.20(1.43,7.18)	REF	1.10(0.47,2.56)	3.48(1.32,9.19)	1.81(0.47,6.92)
P value ^b^		0.866	0.369	**0.005**		0.832	**0.012**	0.386
LGA	68(10.2)	48(16.1)	20(21.5)	12(20.0)	29(12.4)	14(12.0)	11(26.2)	7(31.8)
aOR (95% CI) ^a^	REF	1.70(1.14,2.54)	2.48(1.41,4.36)	2.27(1.14,4.52)	REF	1.04(0.52,2.08)	2.94(1.27,6.81)	3.16(1.14,8.76)
P value ^b^		**0.009**	**0.002**	**0.020**		0.913	**0.012**	**0.027**

Values are presented as frequency (percentage)

^a^ aOR: Odds ratio and 95% confidence interval (CI) were calculated from logistic regression models to reflect the associations between parental prepregnancy BMI and neonatal outcomes of singletons. Adjusted models are controlled for parental age, type of infertility, duration of infertility, ovulatory dysfunction and endometriosis

^b^ *P* value is based on multilevel logistic regression analyses. Results in bold indicate statistical significance (*P* < 0.05)

*BMI* body mass index, *M* maternal prepregnancy BMI, *P* paternal prepregnancy BMI, *IVF* in vitro fertilization, *ICSI* intracytoplasmic sperm injection, *LGA* large for gestational age, *REF* reference group

For twins, the linear regression results showed IVF female newborns from the couples with maternal prepregnancy BMI ≥25 kg/m^2^ had lower birth weight (aB -0.21, 95% CI -0.37-(−)0.04) than both parental BMI < 25 kg/m^2^ (Table S2).

### Birth defect

Among 1366 singletons and 952 twins with information about birth defect, 155(6.69%) children were diagnosed as birth defect. Among all singletons, the prevalence of any birth defect was 6.67% for all IVF/ICSI offspring (*n* = 1366), 6.98% for IVF offspring (*n* = 988) and 5.82% for ICSI offspring (*n* = 378). Regarding twins, the prevalence of any birth defect was 6.72% for all IVF/ICSI offspring (*n* = 952), 7.01% for IVF offspring (*n* = 742) and 5.71% for ICSI offspring (*n* = 210).

### Association between parental prepregnancy BMI and birth defect

Table 4 showed details on the associations between parental prepregnancy BMI and any birth defect. For IVF singletons, couples with paternal prepregnancy BMI ≥25 kg/m^2^ had a trend towards an increased risk of any birth defect (aOR 1.82, 95% CI 1.06–3.10) compared to couples with both parental prepregnancy BMI < 25 kg/m^2^. For ICSI singletons, couples with maternal prepregnancy BMI ≥25 kg/m^2^ had a four-fold higher risk of any birth defect (aOR 4.89, 95% CI 1.45–16.53) compared to couples with both parental prepregnancy BMI < 25 kg/m^2^. No association was seen between parental prepregnancy BMI and any birth defect of twins.

**Table 4:** Associations between parental prepregnancy BMI and any birth defect in multilevel logistic regression analyses

Any birth defect	Reference group				OR (95% CI) ^a^	P value ^b^	aOR (95% CI) ^c^	***P*** value ^b^
Singletons								
IVF	A(M&P<25)	35(5.8)	B(M < 25&*P* ≥ 25)	26(9.8)	1.76(1.04,2.99)	**0.036**	1.82(1.06,3.10)	**0.029**
			C(M ≥ 25&P < 25)	5(7.1)	1.25(0.47,3.30)	0.652	1.23(0.46,3.28)	0.677
			D(M&P ≥ 25)	3(6.3)	1.08(0.32,3.66)	0.897	1.18(0.34,4.04)	0.797
ICSI	A(M&P<25)	8(3.7)	B(M < 25&P ≥ 25)	8(7.2)	2.02(0.74,5.53)	0.172	2.10(0.76,5.84)	0.155
			C(M ≥ 25&P < 25)	5(14.7)	4.48(1.37,14.63)	**0.013**	4.89(1.45,16.53)	**0.011**
			D(M&P ≥ 25)	1(5.9)	1.63(0.19,13.81)	0.657	1.57(0.18,13.60)	0.685
Twins								
IVF	A(M&P<25)	25(5.9)	B(M < 25&P ≥ 25)	22(9.6)	1.69(0.93,3.07)	0.086	1.69(0.92,3.09)	0.089
			C(M ≥ 25&P < 25)	5(8.3)	1.45(0.53,3.95)	0.466	1.73(0.62,4.81)	0.293
			D(M&P ≥ 25)	0	–	–	–	–
ICSI	A(M&P<25)	9(7.1)	B(M < 25&P ≥ 25)	1(1.5)	0.20(0.03,1.61)	0.131	0.21(0.03,1.73)	0.146
			C(M ≥ 25&P < 25)	1(10.0)	1.44(0.16,12.71)	0.740	2.06(0.21,20.51)	0.539
			D(M&P ≥ 25)	1(12.5)	1.86(0.21,16.80)	0.582	0.89(0.09,9.20)	0.925

Values are presented as frequency (percentage)

^a^ Odds ratio (OR) and 95% confidence interval (CI) were calculated from logistic regression models to reflect the association between parental prepregnancy BMI and any birth defect

^b^ *P* value is based on logistic regression analyses. Results in bold indicate statistical significance (*P* < 0.05)

^c^ aOR: Adjusted models are controlled for parental age, type of infertility, duration of infertility, ovulatory dysfunction and endometriosis

*BMI* body mass index, *IVF* in vitro fertilization, *P* paternal prepregnancy BMI, *REF* reference group

### Association between paternal prepregnancy BMI and birth defect

All singletons conceived via IVF were further grouped based on paternal prepregnancy BMI as follows: 674(68.2%) singletons with paternal prepregnancy BMI < 25 kg/m^2^ and 314(31.8%) singletons with paternal prepregnancy BMI ≥25 kg/m^2^. Table 5 included details on the association between paternal prepregnancy BMI and birth defect of IVF singletons. We found that couples with paternal prepregnancy BMI ≥25 kg/m^2^ had a four-fold increased risk of congenital malformations of the musculoskeletal system (aOR 4.38, 95% CI 1.31–14.65) compared to couples with paternal prepregnancy BMI < 25 kg/m^2^. This association still remained after adjustment for confounding factors (aOR 4.55, 95% CI 1.32–15.71). No association was seen between paternal prepregnancy BMI and risk of other subcategories of birth defects.

**Table 5:** Associations between paternal prepregnancy BMI and birth defect of IVF singletons in logistic regression analyses

The subcategories of birth defect	BMI(kg/m^**2**^)		OR (95% CI) ^a^	P value ^b^	aOR (95% CI) ^c^	***P*** value ^b^
	P<25 (***n*** = 674)	P ≥ 25 (***n*** = 314)				
Multiple birth defects	2(0.3)	1(0.3)	1.07(0.10,11.88)	0.954	0.62(0.04,10.12)	0.736
The nervous system	5(0.7)	3(1.0)	1.29(0.31,5.44)	0.728	1.38(0.32,5.99)	0.665
Eye, ear, face and neck	5(0.7)	6(1.9)	2.61(0.79,8.61)	0.116	2.73(0.82,9.16)	0.103
The circulatory system	11(1.6)	4(1.3)	0.78(0.25,2.46)	0.669	0.79(0.25,2.55)	0.697
The digestive system	3(0.4)	1(0.3)	0.72(0.07,6.90)	0.771	0.78(0.08,7.62)	0.831
The genital organs	2(0.3)	0	–	–	–	–
The urinary system	6(0.9)	1(0.3)	0.36(0.04,2.97)	0.340	0.45(0.05,3.84)	0.468
The musculoskeletal system	4(0.6)	8(2.5)	4.38(1.31,14.65)	**0.017**	4.55(1.32,15.71)	**0.016**
Chromosomal abnormalities	4(0.6)	2(0.6)	1.07(0.20,5.89)	0.935	1.03(0.18,5.90)	0.976
Other congenital malformations	3(0.4)	5(1.6)	3.62(0.86,15.24)	0.080	3.07(0.71,13.23)	0.132

Values are presented as frequency (percentage)

^a^ Odds ratio (OR) and 95% confidence interval (CI) were calculated from logistic regression models to reflect the association between paternal prepregnancy BMI and the subcategories of birth defect. Couples with paternal prepregnancy BMI < 25 kg/m^2^ as reference group

^b^ P value is based on logistic regression analyses. Results in bold indicate statistical significance (*P* < 0.05)

^c^ aOR: Adjusted models are controlled for parental age, maternal prepregnancy BMI, type of infertility, duration of infertility, ovulatory dysfunction and endometriosis

*BMI* body mass index, *IVF* in vitro fertilization, *P* paternal prepregnancy BMI

## Discussion

As risk factors of poor reproductive condition, overweight and obesity are likely more common in infertile couples. Compared with spontaneous pregnancy, couples seeking ART treatments are more concerned about the live birth rate and the health of offspring. Our study explored the effect of parental prepregnancy BMI on the neonatal outcomes and birth defect in fresh autologous cycles, which may offer advice for improving the effectiveness of ART treatment.

Despite no significant differences in the number of oocytes retrieved, 2PN rate and clinical pregnancy rate among four groups, maternal prepregnancy BMI ≥25 kg/m^2^ was associated with lower live birth rate in IVF cycles. This finding is consistent with several previous published studies [23–25]. However, no significant differences were observed in the live birth rate of ICSI cycles among four groups, which is consistent with prior studies. A meta-analysis by Le et al. suggested that increased male BMI could hardly affect the live birth rate of ICSI treatment [26]. Ozgun et al. also found no significant difference in live birth rate between women with normal-weight and obesity underwent ICSI treatment [27].

After analyzing the neonatal outcomes of 1534 singletons, we observed that the proportion of LGA is significantly different across four groups in both IVF and ICSI cycles, especially the sizable increase in group C and group D. It suggested that increased parental prepregnancy BMI is a risk factor of LGA. This association has been partly found in previous studies. A study by Anzola et al. concluded that the percentage of LGA was influenced by a high maternal BMI in IVF-FET cycles [28]. A retrospective analysis of 12,950 deliveries, which didn’t restrict the study population to offspring conceived via ART, investigated that women classified as obesity before pregnancy were at increased risk for LGA (16.8% vs 10.5%, OR 1.72, 95%CI 1.57–1.97) [29]. The underlying mechanism could be in part explained by insulin resistance in women with obesity which resulted in the increased nutrients supply to the fetus through the placenta [30]. The levels of tumor necrosis factor α (TNF- α) in cord blood are higher in obese women, which is known associated with an increased risk of LGA [31]. However, there is little research on the relationship between LGA and male prepregnancy BMI in ART cycles. Although the precise mechanisms for the effect of paternal prepregnancy BMI on LGA remain unknown, it is likely that epigenetic changes such the expression of sperm microRNAs, histone modification and DNA methylation in spermatozoa caused by paternal overweight or obesity induce the fetal overgrowth [32, 33]. Much additional research should be conducted to confirm our findings.

In this study, parental overweight or obesity was associated with the higher odds of fetal macrosomia. In the meanwhile, the incidence of cesarean section markedly increased with maternal prepregnancy BMI ≥25 kg/m^2^. Those findings are consistent with previous studies demonstrating higher incidence of fetal macrosomia and cesarean section with raised female BMI underwent IVF/ICSI treatment [10, 34–36]. Interestingly, our study showed lower proportion of male newborns with increased maternal BMI. Moreover, parental prepregnancy overweight or obesity strongly affects the birth weight of female newborns and has no significant effect on male birth weight. In previous studies, sex ratio biased towards female infants had been found in women with low prepregnancy BMI and smoking or aged parents [37–39]. Women with metabolic alterations, such as diabetes, also accounted for the lower sex ratio [40]. The mechanisms of the different impact of parental overweight or obesity on male and female newborns was not clarified, which should be investigated by more researches in the future.

Regarding the neonatal outcomes of twins, only the birth weight of female infants significantly decreased for couples with maternal prepregnancy BMI ≥ 25 kg/m^2^ compared with group A. Other associations between the neonatal outcomes and parental prepregnancy BMI which we found in singletons were disappeared in twins. The differences of outcomes between the singletons and twins had been discussed in several previous studies. A retrospective population-based study in Canada showed that the association of maternal obesity with adverse pregnancy outcomes in twins is weaker than that observed in singletons [41]. Another research of 12,061 first fresh IVF/ICSI cycles indicated that the birth weight of singletons was significantly higher when parental BMI were greater, but no significant differences were observed in twins [9]. The insignificant effect of parental prepregnancy BMI on the neonatal outcomes in twins may be explained by the higher risk of complications in multiple pregnancy which may override the influence of parental obesity [42–44].

Birth defect is the most important outcome in our study. The prevalence of any birth defect was 6.69% for all births in our study. It was higher than the birth defects rate (5.6%) published by National Health Commission of China in 2012. On the one hand, many factors which are common in infertile population may improve the risk of birth defects, such as advanced age, environment pollution exposure and genetic factors [14, 45, 46]. On the other hand, the procedures of ART may be associated with a higher risk of birth defects compared with spontaneous pregnancy [47, 48]. The prevalence of any birth defect was 8.3% in assisted conception and 5.8% in spontaneous conception according to a research of 308,974 births in South Australia [48].

In our study, we observed that parental prepregnancy BMI ≥25 kg/m2 was associated with a higher risk of any birth defect in IVF/ICSI offspring. Some published studies have explored the association between maternal BMI and different subcategories of birth defects. A meta-analysis of 18 studies investigated that women with obesity were more likely to an infant with neural tube defects (OR 1.87, 95% CI 1.62–2.15), cardiovascular anomalies (OR 1.30, 95% CI 1.12–1.51) and other structural anomalies than mothers with normal BMI [17]. Another research showed that mothers with obesity had an overall increased risk for having an infant with orofacial clefts [49]. However, there was limited evidence showing the association between the paternal prepregnancy BMI and birth defects before. A cohort study conducted by National Research Institute for Family Planning in China observed that the ORs of birth defect for men with overweight and obesity versus men with normal BMI was 1.12 (95%CI 0.99–1.28) and 1.32 (95%CI 1.05–1.64). They also found that couples with overweight or obesity had higher odds of adverse pregnancy outcomes than couples with normal BMI [50]. Van et al. found no significant association between increased paternal prepregnancy BMI and anorectal malformation [51]. Nevertheless, those studies did not distinguish between spontaneous pregnancy and assisted pregnancy. To our knowledge, our study provides the first measurements of the association between birth defects of offspring conceived via IVF/ICSI and increased paternal prepregnancy BMI.

On further subcategory analysis, we found that paternal overweight or obesity was associated with a higher risk of congenital malformations of musculoskeletal system. A rat model study proposed that impaired muscle growth at 8 wk. of age in offspring from obese fathers is driven by a decrease in GH secretion and IGF-I level [52]. Some studies had indicated that paternal obesity can reduce skeletal muscle insulin sensitivity [53, 54]. No previous research had investigated the effect of paternal obesity on the fetal skeletal muscle development. Thus, additional studies are needed to determine the underlying mechanisms responsible for such an association.

There are some limitations in our study. One is that the sample size was small, especially of the subgroup of twins or ICSI. It may prevent us from accurately assessing the impact of increased parental prepregnancy BMI on neonatal outcomes and birth defects. Another limitation is that the study is retrospective cohort study. Part of the information about birth defects was collected by nurse via telephone, which may introduce recall bias. We tried to reduce risk of bias by explaining in detail the definition of birth defects and repeatedly confirming whether there is a hospital diagnosis certificate. Additionally, there is little research on the relationship between paternal prepregnancy BMI and birth defects of offspring after IVF/ICSI at present. More studies about this issue must be conducted in the future.

## Conclusion

In summary, our study indicates that parental prepregnancy overweight or obesity has adverse effect on the neonatal outcomes and birth defect in fresh autologous cycles. Increased parental prepregnancy BMI, independently or combined, improves the odds of LGA and birth defect in singletons. For couples underwent IVF treatments, paternal prepregnancy BMI ≥25 kg/m^2^ have higher risk of the incidence of congenital malformations of the musculoskeletal system. As a rare study investigating the effect of paternal prepregnancy BMI on the birth defects, our study could serve as a reference for further research. According to findings from our study and previous studies, couples planning to get pregnant should be advised to control their weight before pregnancy. Strengthening public health education about balanced diet and healthy lifestyle may contribute to the prevention of birth defect.

## Supplementary Information

**Additional file 1: Table S1**. The cycle outcomes compared by maternal and paternal prepregnancy BMI.

**Additional file 2: Table S2**. Associations between parental prepregnancy BMI and birth weight of IVF female newborns in multilevel linear regression analyses.

## Data Availability

The datasets used and/or analyzed during the current study are available from the corresponding author on reasonable request.

## Abbreviations

BMI: Body mass index
IVF: In vitro fertilization
ICSI: Intracytoplasmic sperm injection
ART: Assisted reproductive technology
SGA: Small for gestational age
LGA: Large for gestational age
2PN: Two pronuclear
SD: Standard deviation
CIs: Confidence intervals
OR: Odds ratio
aOR: Adjusted odds ratio
