# Supplementary material for: Association of parental prepregnancy BMI with neonatal outcomes and birth defect in fresh embryo transfer cycles: a retrospective cohort study
Source: BMC Pregnancy Childbirth. 2021 Nov 27;21:793. doi: 10.1186/s12884-021-04261-y (PMC8627045; doi:10.1186/s12884-021-04261-y)
Supplement: Supplementary file 1 — Additional file 1: Table S1. The cycle outcomes compared by maternal and paternal prepregnancy BMI. [file 12884_2021_4261_MOESM1_ESM.docx]

**Table S1 The cycle outcomes compared by maternal and paternal prepregnancy BMI**

|  | **IVF** | | | | | **ICSI** | | | | |
| --- | --- | --- | --- | --- | --- | --- | --- | --- | --- | --- |
| **Variable** | **A(M&P<25)** | **B(M<25&P≥25)** | **C(M≥25&P<25)** | **D(M&P≥25)** | **P value ^a^** | **A(M&P<25)** | **B(M<25&P≥25)** | **C(M≥25&P<25)** | **D(M&P≥25)** | **P value ^a^** |
| No. of cycles | 2346 | 1152 | 412 | 265 |  | 930 | 419 | 121 | 96 |  |
| No. of oocytes retrieved | 10.66±5.29 | 10.34±5.49 | 10.57±5.58 | 9.99±5.39 | 0.147 | 10.89±5.26 | 10.79±5.45 | 11.36±5.65 | 10.30±5.00 | 0.523 |
| 2pn rate (%) | 59.47±23.65 | 60.76±24.31 | 59.32±24.03 | 57.99±24.19 | 0.267 | 47.18±19.97 | 48.09±20.75 | 49.23±21.16 | 44.45±19.44 | 0.305 |
| No. of embryos transferred | 1.87±0.54 | 1.88±0.52 | 1.89±0.53 | 1.91±0.57 | 0.626 | 1.83±0.55 | 1.82±0.58 | 1.85±0.56 | 1.85±0.56 | 0.928 |
| Clinical pregnancy | 1072(45.7) | 518(45.0) | 162(39.3) | 118(44.5) | 0.123 | 380(40.9) | 185(44.2) | 58(47.9) | 34(35.4) | 0.191 |
| Live birth | 894(38.1) | 419(36.4) | 130(31.6) ^c^ | 84(31.7) ^b^ | **0.022** | 307(33.0) | 151(36.0) | 47(38.8) | 26(27.1) | 0.213 |

Values are presented as mean ± SD or frequency (percentage).

^a^ P value is based on One-Way ANOVA for continuous variables and χ2 test for categorical variables across maternal and paternal prepregnancy BMI. Results in bold indicate statistical significance (P < 0.05).

^b^ Significant difference was found compared with group A using regression models (linear or logistic as appropriate) without adjusting for confounders.

^c^ Significant difference was found compared with group A using regression models (linear or logistic as appropriate) with adjusting for parental age, type of infertility, duration of infertility, ovulatory dysfunction and endometriosis.

*BMI* body mass index, *M* maternal prepregnancy BMI, *P* paternal prepregnancy BMI, *IVF* in vitro fertilization, *ICSI* intracytoplasmic sperm injection, *2pn* two pronuclear
