# Supplementary material for: Association of parental prepregnancy BMI with neonatal outcomes and birth defect in fresh embryo transfer cycles: a retrospective cohort study
Source: BMC Pregnancy Childbirth. 2021 Nov 27;21:793. doi: 10.1186/s12884-021-04261-y (PMC8627045; doi:10.1186/s12884-021-04261-y)
Supplement: Supplementary file 2 — Additional file 2: Table S2. Associations between parental prepregnancy BMI and birth weight of IVF female newborns in multilevel linear regression analyses. [file 12884_2021_4261_MOESM2_ESM.docx]

**Table S2 Associations between parental prepregnancy BMI and birth weight of IVF female newborns in multilevel linear regression analyses**

|  | **Singletons** | | | | **Twins** | | | |
| --- | --- | --- | --- | --- | --- | --- | --- | --- |
| **Variable** | **A(M&P<25)** | **B(M<25&P≥25)** | **C(M≥25&P<25)** | **D(M&P≥25)** | **A(M&P<25)** | **B(M<25&P≥25)** | **C(M≥25&P<25)** | **D(M&P≥25)** |
| Birth weight (kg, girl) | 3.17±0.42 | 3.25±0.48 | 3.30±0.56 | 3.35±0.45 | 2.41±0.43 | 2.31±0.48 | 2.20±0.49 | 2.36±0.60 |
| aB (95% CI) ^a^ | REF | 0.08(-0.01,0.18) | 0.14(0.01,0.27) | 0.20(0.03,0.36) | REF | -0.10(-0.21,0.00) | -0.21(-0.37, -0.04) | -0.06(-0.26,0.14) |
| P value ^b^ |  | 0.083 | **0.040** | **0.020** |  | 0.056 | **0.015** | 0.564 |

Values are presented as mean ± SD.

^a^ aB: Coefficient (B) and 95% confidence interval (CI) were calculated from linear regression models to reflect the relationship between parental prepregnancy BMI and birth weight of IVF female newborns. Adjusted models are controlled for parental age, type of infertility, duration of infertility, ovulatory dysfunction and endometriosis.

^b^ P value is based on multilevel linear regression analyses. Results in bold indicate statistical significance (P < 0.05).

*BMI* body mass index, *M* maternal prepregnancy BMI, *P* paternal prepregnancy BMI, *IVF* in vitro fertilization, *REF* reference group
